# Editorial: Energy Requirements in Membrane Trafficking

**DOI:** 10.3389/fcell.2021.750633

**Published:** 2021-10-14

**Authors:** Carlos M. Guardia, Aitor Hierro, David C. Gershlick

**Affiliations:** ^1^Section on Intracellular Protein Trafficking, Neurosciences and Cellular and Structural Biology Division, Eunice Kennedy Shriver National Institute of Child Health and Human Development, National Institutes of Health, Bethesda, MD, United States; ^2^CIC bioGUNE, Basque Research and Technology Alliance (BRTA), Bizkaia Technology Park, Derio, Spain; ^3^Ikerbasque, Basque Foundation for Science, Maria Diaz de Haro, Bilbao, Spain; ^4^Cambridge Institute for Medical Research, University of Cambridge, Cambridge, United Kingdom

**Keywords:** ATP, GTP, small GTPase, motor molecules, membranes

Whilst studying pancreatic exocrine cells in the 1960's, George Palade discovered that intracellular trafficking events require energy (Jamieson and Palade, 1968), a finding he described as “unexpected” in his Nobel prize lecture (Palade, 1974). The fundamental membrane trafficking components were subsequently determined by the labs of James Rothman, Randy Schekman, and others (Novick and Schekman, 1979; Balch et al., 1984; Kaiser and Schekman, 1990; Perin et al., 1990), inaugurating the modern era of intracellular pathways research. One of those key components was NSF (*N*-ethylmaleimide sensitive factor) initially purified from an *in vitro* reconstitution of Golgi membranes (Block et al., 1988). NSF was later shown to be the ATPase essential for the disassembly of SNARE complexes which mediate membrane fusion (Hata et al., 1993; Söllner et al., 1993), and thus the fundamental principle of subcellular trafficking events requiring energy was established.

One of the most remarkable characteristics of living cells is their ability to adapt and self-organize in response to environmental changes making them out-of-equilibrium systems. This dynamic equilibrium tries to maintain the internal environment of the cell as stable as possible *via* energy transformation. Through intracellular membrane trafficking and complex metabolic pathways, eukaryotic cells have evolved to efficiently transform the energy from their environment into energy storage molecules like ATP, GTP, NADH, and acetyl-CoA. Most of the energy is used to synthesize the biomolecules that provide cells with the tools to survive and interact with their environment (Traut, 1994; Walsh et al., 2018). A great part of the remaining energy produced is used to move the cell around, support cell division and differentiation, incorporate material from the extracellular space, secrete signaling products, assure protein homeostasis, transport cargo along the interior of the cell, and recycle material when necessary (Zala et al., 2017). These processes are therefore exquisitely regulated by events where the limiting step requires the release of energy by ATP or GTP in a controlled manner.

In particular, a number of energy-dependent proteins can be found to be essential for all the steps involved in the intracellular traffic of cargo. This Research Topic contains 10 articles that explore a variety of energy requiring processes in the cell.

At the border of the cell, the intracellular journey of a cargo starts with its internalization by endocytosis. In that context, Matthaeus and Taraska summarize the recent concepts in caveolae trafficking and dynamics with a focus on the ATP and GTP-regulated proteins that control caveolae behavior and their role in the cell metabolism. Once the cargo reaches the endo-lysosomal system, many factors and signals can determine its next destination. Saric and Freeman discuss how controlled solute efflux along the endocytic pathway contributes to membrane tension regulation and membrane remodeling with special emphasis on lysosomal solute transport. Wang and Ye review recent exciting studies on novel translocon-associated quality control strategies that cells use for eliminating polypeptides trapped in the endoplasmic reticulum and mitochondria during *de novo* protein synthesis.

Small GTPases act as signaling components that regulate subcellular processes and define sub-cellular compartments. GTP-bound GTPases have the power to recruit effectors that help shape and regulate these organelles and endosomes. Adarska et al. present a summary of the often-overlooked important roles of the ARF1-5 GTPases in trafficking while Fujibayashi and Mima cover a novel role for ARF6 as a membrane tether using chemically defined reconstitution approach with purified proteins and liposomes.

Relatively large cargo (e.g., vesicles, organelles like endosomes, autophagosomes, mitochondria, or molecules such as mRNA-protein complexes) needs to be transported around the cell by the help of motor molecules such as kinesins and dyneins. These molecular machines use the energy of ATP hydrolysis to generate processive and efficient distribution of cargo and their carriers along the microtubule network of the cell. A set of comprehensive reviews from Yadav and Kunwar and Xiang and Qiu summarize our understanding of the effect of temperature on motor molecules and the cargo-mediated activation of cytosolic dynein, respectively, and address important questions whose answers will push forward the research on the growing field of intracellular transport and dynamics.

Finally, this Research Topic includes three insightful articles that put the role of intracellular trafficking in the context of disease. Schiavon et al. discuss previous work on mitochondria intracellular transport and provide an example where impaired mobility of mitochondria may be playing a central role in the pathogenesis of Charcot-Marie-Tooth (CMT) disease, a neurodegenerative disorder. Additionally, Sneeggen et al. describe how the role of intracellular trafficking in cell proliferation, epithelial to mesenchymal transition, and invasion contributes to the regulation of energy consumption and metabolism during cancer progression. Lastly, Tavares et al. delineate the capacity of the HIV-1 virus to hijack key machinery of the intracellular pathway in order to ensure efficient viral replication and survival in the host.

Altogether, these contributions should give the reader an updated view of different aspects of the intracellular traffic field and a glimpse on the future directions of research in each sub-field. We hope the reader can also find the gaps and the many still unanswered questions in the field while navigating through this Research Topic: how is the motor molecules' activity regulated during development and disease progression? What is the next technology that will thrust this field into the next century of discovery? At the molecular level, how do we modulate cargo localization through targeting strategies, and in doing so, influence the adaptive requirements of a cell? How can we prevent future public health crises by manipulating the host-microorganism energy supply and demand flux? How can we generate a more comprehensive understanding of the molecular machinery of protein trafficking? The dysregulation of the machinery involved in controlling intracellular traffic is frequently associated with diseases that include cancer, developmental and degenerative diseases, and multiple immunity disorders, highlighting the urgency of unraveling the mechanisms that regulate the movement of cargo inside the cell.

## Author Contributions

All authors listed have made a substantial, direct and intellectual contribution to the work, and approved it for publication.

## Funding

AH was funded by the Spanish Ministry of Economy and Competitiveness (BFU2017-88766-R and PID2020-119132GB-I00). DCG was funded by a Sir Henry Dale Fellowship awarded from the Wellcome Trust/Royal Society (Grant No. 210481).

## Conflict of Interest

The authors declare that the research was conducted in the absence of any commercial or financial relationships that could be construed as a potential conflict of interest.

## Publisher's Note

All claims expressed in this article are solely those of the authors and do not necessarily represent those of their affiliated organizations, or those of the publisher, the editors and the reviewers. Any product that may be evaluated in this article, or claim that may be made by its manufacturer, is not guaranteed or endorsed by the publisher.
